# Effect of Spray Dryer Scale Size on the Properties of Dried Beetroot Juice

**DOI:** 10.3390/molecules26216700

**Published:** 2021-11-05

**Authors:** Jolanta Gawałek

**Affiliations:** Department of Dairy and Process Engineering, Poznań University of Life Sciences, 60-624 Poznań, Poland; jolanta.gawalek@up.poznan.pl

**Keywords:** spray drying, vegetable powders, beetroot, natural colorants, violet betalain pigments, polyphenols

## Abstract

Experiments detailing the spray drying of fruit and vegetable juices are necessary at the experimental scale in order to determine the optimum drying conditions and to select the most appropriate carriers and solution formulations for drying on the industrial scale. In this study, the spray-drying process of beetroot juice concentrate on a maltodextrin carrier was analyzed at different dryer scales: mini-laboratory (ML), semi-technical (ST), small industrial (SI), and large industrial (LI). Selected physicochemical properties of the beetroot powders that were obtained (size and microstructure of the powder particles, loose and tapped bulk density, powder flowability, moisture, water activity, violet betalain, and polyphenol content) and their drying efficiencies were determined. Spray drying with the same process parameters but at a larger scale makes it possible to obtain beetroot powders with a larger particle size, better flowability, a color that is more shifted towards red and blue, and a higher retention of violet betalain pigments and polyphenols. As the size of the spray dryer increases, the efficiency of the process expressed in powder yield also increases. To obtain a drying efficiency >90% on an industrial scale, process conditions should be selected to obtain an efficiency of a min. of 50% at the laboratory scale or 80% at the semi-technical scale. Designing the industrial process for spray dryers with a centrifugal atomization system is definitely more effective at the semi-technical scale with the same atomization system than it is at laboratory scale with a two-fluid nozzle.

## 1. Introduction

The food market is subject to constant changes, which results, among others, from the influence of current global trends and greater consumer knowledge and awareness, as well as changes in the behaviors and lifestyles of society. Low-quality goods containing large amounts of flavor enhancers, preservatives, or artificial colorants are becoming less and less popular. Another trend, resulting from the lifestyle of a wide range of people, is the increased popularity of foods that are convenient for quick preparation, including instant foods. In this type of food, spray-dried fruit and vegetable powders are gaining popularity as ingredients in final food products. These act as carriers for flavor, aroma, and color and also have bioactive properties. One of the most important examples of these carriers is spray dried beetroot (*Beta vulgaris*) juice, which is the main ingredient in many instant soups but also in a wide range of food products, where it plays the role of a natural colorant. The development of instant meals in the global market has been characterized by very dynamic growth in recent years. It manifests itself as a huge increase in diversity, both in terms of taste and in the form of administration and packaging. As a result, the ingredients of such foods are subject to increasingly higher quality requirements so that they can meet the higher technological demands for the production of attractive, natural, and healthy instant foods. The use of a given food ingredient in the final product usually involves the precise design of its properties, and this, in turn, requires a well-designed spray drying process for that ingredient.

Spray drying is the most widely used technique for liquid food drying and encapsulation. The advantage of this method is related to its economy, flexibility, and possibility of continuous operation [1,2,3]. It allows the stability of the product to be increased, reducing volume of the product and extending its shelf life, which also facilitates product handling and also allows products to be stored at ambient temperature [1,3,4,5]. When considering the spray drying of fruit and vegetable juices, there are technological problems that are associated with the hygroscopic and thermoplastic properties of the juices (product sticking and settling on the walls of the dryer, reduction of drying efficiency and powder stability, operational problems of the dryer) [6,7,8]. The reason for this is the content of the organic acids and low molecular weight sugars with a low glass transition temperature that are contained in the fruit and vegetable juices [9,10]. A known way to cope with these difficulties is to add drying carriers that have high molecular weight and that can effectively lower the glass transition temperature of the dried juice [11]. The most commonly used carriers in spray drying processes are polysaccharides, proteins, and lipids [12,13,14]. For fruit and vegetable juice drying, polysaccharides such as hydrolyzed starches or gums are the most preferred due to their chemical similarity and plant origin but also because of their lower purchase cost compared to other carriers.

Designing the beetroot juice spray drying process and the specific properties of the resulting beetroot powder comes down to the optimal selection of the main raw material—beetroot juice concentrate, the selection of the carrier, and the selection of appropriate drying process conditions. This is often first completed on a laboratory and/or semi-technical scale, and the drying is then transferred to an industrial scale of an appropriate size, depending on the planned production batch. When designing a new industrial dryer for a specific product, laboratory tests are necessary to acquire the needed information about the behavior of the dried materials [7]. The laboratory and semi-technical scale tests performed help to design the industrial process. However, differences in product quality at different scales present some difficulties in the development and production of powder products [15]. This is largely due to the fact that spray drying involves a combination of complex physical transformations that include the mixing of the main dried component and excipients in a suitable solvent, liquid atomization, solvent evaporation (drying), gas–particle separation, and often secondary drying, which may take place in a fluidized bed dryer [16]. Scale-up studies of spray dryers (particularly in the field of pharmaceutical drying) can be found in the literature and include specific process scaling-up design procedures [15,16,17,18,19,20,21]. However, the limited number of literature reports on industrial scale spray drying is due to the high cost of conducting such experiments and validating theoretical models [21].

This work focuses on the determination and comparison of the physicochemical properties of dried beetroot powders that were obtained in dryers of different scales, ranging from the laboratory to large industrial scales. Determining the trends of changes in individual functional properties for this type of product (natural colorants of plant origin) will enable more the precise planning of production processes for different production batches. As mentioned above, the quality requirements for such powdered food ingredients are very high; in particular, repeatability is very important. A given product must be functionally the same regardless of whether it is produced in a large or small production batch. Additionally, it is known that the most economical drying takes place when the appropriate batch size is matched to the corresponding size of the spray dryer.

## 2. Results and Discussion

### 2.1. Powder Yield

**Figure 1:**
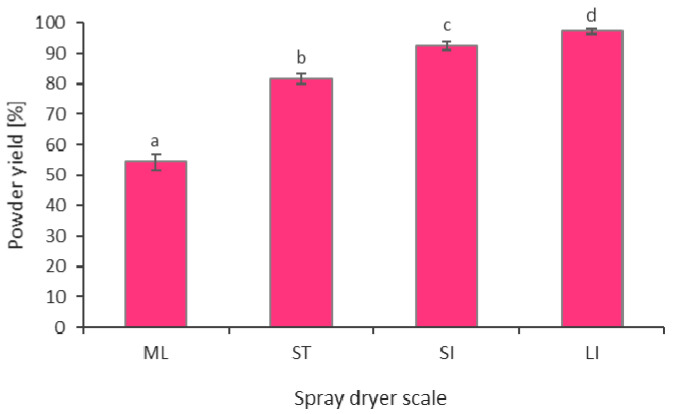
Powder yield of spray dried beetroot juice at different process scales (ML—mini-laboratory scale, ST—semi-technical scale, SI—small industrial scale, LI—large industrial scale; a, b, c, d—different letters show significant differences between spray dryer scale (*p* ≤ 0.05)).

Powder yield is a very important indicator that is related to the efficiency and economy of the production process. Designing an industrial spray drying process requires achieving a high powder yield that is at least above 90% for the production to make economic sense, and preferably, the production yield is above 97%. In other words, carriers, recipes, and drying conditions should be selected in such a way so as to minimize product losses to at least below 10%, and preferably below 3%. The determined powder yields for the conducted beetroot juice spray drying processes at different scales are presented in Figure 1. The differences in the values for individual spray dryers show statistical significance, with the larger spray-drying scale allowing for significantly higher powder yields. For both industrial-scale dryers (SI and LI), powder yields above 90% were achieved, which indicates well-chosen drying conditions for the studied beetroot juice concentrate. The powder yield values for the experimental driers on the laboratory (ML) and semi-technical (ST) scales are much lower, i.e., 54.3 and 81.6%, respectively. Other researchers who conducted research on laboratory-scale beetroot juice achieved powder yield values in the ranges of 15–18% (Poornima et al. [22]), 41.31–54.63% (Bazaria et al. [23]), and 41.33–56.55% (Teenu et al. [24]). Jedlińska et al. [25] conducted a semi-technical-scale experiment in which they spray-dried cloudy beetroot juice with dehumidified air at low inlet temperatures (90 °C and 130 °C). The temperature of 130 °C resulted in a powder yield of 76.1% and 90%, with and without maltodextrin as a carrier, respectively. However, it is noteworthy that the feed solutions in this study had a much lower concentration, i.e., 13.3 °Bx with the carrier and 8 °Bx without the carrier. As seen from the study results, the semi-technical scale definitely approximates (simulates) the industrial process better, which is also confirmed by the above-mentioned studies from other authors.

From this analysis of the powder yield changes, the values that must be achieved at the experimental scales to obtain satisfactory spray drying efficiency at industrial scales. It can be assumed that for the laboratory scale, it is a min. of 50%, and for the semi-technical scale, the min. is 80%. Achieving such drying effectiveness at the experimental scale will enable the transfer of the determined optimum drying conditions to the industrial scale, allowing the production effectiveness indicators of >90% to be achieved.

### 2.2. Microstructure of Particles

**Figure 2:**
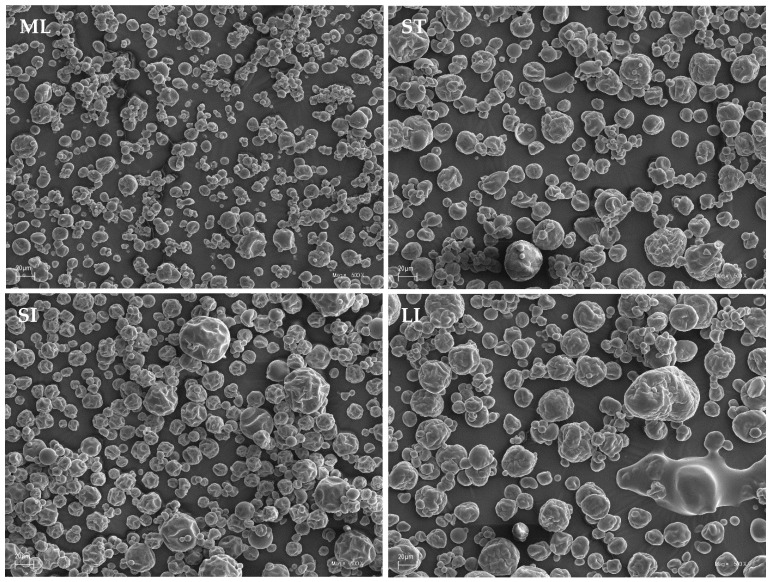
Microstructure of spray-dried beetroot juice particles (magnification 500×) at different process scales (ML—mini-laboratory scale, ST—semi-technical scale, SI—small industrial scale, LI—large industrial scale).

**Figure 3:**
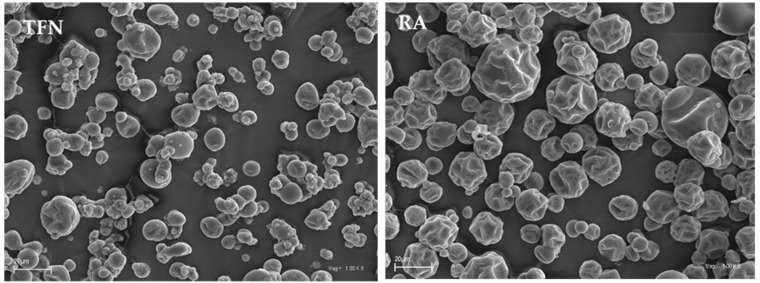
A comparison of particle morphology of two different liquid spray systems (magnification 1000×): two-fluid nozzle (TFN) and rotary atomizer (RA).

Figure 2 shows microphotographs of beetroot powder particles for different spray drying-scale sizes. The resulting particles are of various sizes and spherical shapes and have numerous folds and cracks, which are typical for crop products that have been spray-dried on maltodextrin carriers. The microstructure of the particles for the three dryers with a centrifugal liquid atomization system (ST, SI, LI) shows no significant differences, while the microstructure of the beetroot powder particles produced in the laboratory dryer (ML) is mildly different. In the first case, more cracks and wrinkles can be observed on the surface. This is related to the large loss of moisture from the particles and their subsequent cooling [25,26]. The use of the laboratory dryer (ML) resulted in much smaller but more spherical and smoother particles. This may have been caused by a different atomization system: a two-fluid nozzle, where the dried liquid is atomized by a stream of compressed air, is conducive to the formation of more small particles. The more developed evaporation surface favours a less violent exit of the contained moisture through the particle surface, which reduces surface shrinkage. Figure 3 compares the particle morphology of two different atomization systems (two-fluid nozzle and centrifugal liquid atomization) at a higher magnification (1000×).

Jedlińska et al. [25] obtained particles with similar morphology. The researchers conducted a semi-technical-scale experiment in which they also spray-dried beet juice with a centrifugal liquid atomization system. The procedure resulted in smaller particles and fewer shrunken particles than when the ST, SI, and LI dryers were used due to the higher rotary atomizer speed, lower carrier content, and lower air inlet temperature than the ones used in our study. Janiszewska [27] also dried beetroot juice with a rotary atomizer on a semi-technical scale. The researcher used a higher content of maltodextrin as a carrier and a higher temperature (the drying conditions were closer to the parameters of our study). The microstructure of the particles was very similar to the microstructures obtained with the ST, SI, and LI spray dryers.

Singh and Hathan [26] analyzed the microstructure of the spray-dried beetroot juice particles obtained with a two-fluid nozzle laboratory dryer. The particles obtained in this study were similar in size and exhibited a similar tendency for agglomeration but had much greater shrinkage than the particles obtained with the ML dryer. The difference may have been caused the higher carrier content.

### 2.3. Particle Size Distribution

The particle size of the beetroot powder obtained by spray drying is a key parameter that affects several of the powders’ functional properties (e.g., bulk density, flowability, compressibility, solubility, and hygroscopicity) [28]. The quantities characterizing the cumulative volumetric particle size distribution are summarized in Table 1. The determined mean values of particle size D[4,3] ranged from 11.8–43.2 µm and increased with increasing dryer size. However, the particle size of the beetroot powder obtained in the laboratory dryer (ML) was much smaller (11.8 µm) than the others (36.1–43.2 µm). Furthermore, the span of the particle size distribution for the laboratory dryer (ML) was much larger (span = 3.03) compared to the other dryers that were tested (ST, SI, LI), which achieved span values in the range of 1.93–2.19. The markedly different nature of the particle size distribution for the ML dryer may not only be due to the much smaller size but also due to the different liquid atomization system. In this case, liquid atomization is performed in a nozzle system, while in the other dryers, it is performed in a centrifugal rotary atomizer system. Janiszewska et al. [27,29] conducted a semi-technical-scale experiment with centrifugal liquid atomization. The researchers used similar amounts of maltodextrin as a carrier in beetroot juice and obtained much smaller particle sizes (9.36–12.81 µm), but they used a much higher rotary atomizer speed (39,000 rpm). Jedlińska et al. [25] used a lower rotary atomizer speed (26,500 rpm). The size of the beetroot juice particles that were spray-dried with maltodextrin was 9.1 µm, whereas the size of the particles spray-dried without maltodextrin ranged from 13.2 to 15.6 µm.

### 2.4. Moisture Content and Water Activity

All of the beetroot powders that were obtained were characterized by low values of both moisture content (2.84–3.10%) and water activity (0.18–0.23). This indicates that adequate microbiological stability was achieved for the beetroot powders obtained in all of the tested drying scales. Among the centrifugal spray dryers (ST, SI, LI), a gentle decreasing trend in the moisture content and in the water activity of the beetroot powders can be observed with the increasing dryer size.

A comparison of the results showed that the increase in the size of the particles was correlated with a simultaneous decrease in the moisture content and water activity in spray-dried beetroot powders. This finding was in line with the typical trends observed in various studies with the same dryer but with an increase in the inlet air temperature (e.g., spray drying of orange juice by Chegini et al. [30]). In our study, the inlet temperature was the same for all of the spray-drying scales. Therefore, this decreasing trend of changes in the moisture content and water activity in beetroot powders along with the increase in the dryer scale should be associated with the decrease in the ratio of the drying air flow to the dryer volume (according to the data in Table 4). This resulted in the beetroot juice having a longer residence time in the drying chamber and a consequently smaller amount of residual moisture in the obtained beetroot powder.

Other researchers have obtained beetroot powder moisture values of 1–3.4% (Poornima et al. [22]), 3.5–6.9% (Jedlińska et al. [25]), 3.95–6.50% (Singh et al. [26]), 2.8–4.5 (Janiszewska [27]), 2.77–4.75% (Janiszewska et al. [29]), and 3.33–4.24% (Do Carmo et al. [31]), and Janiszewska [27] obtained water activity at the level of 0.44–0.75. Jedlińska et al. [25] observed much lower water activity in beetroot powders that had been spray-dried with carriers (0.107–0.148) and without carriers (0.169–0.202). Such low values may have been caused by the use of dehumidified drying air.

### 2.5. Bulk Density, Angle of Repose and Flowability

**Figure 4:**
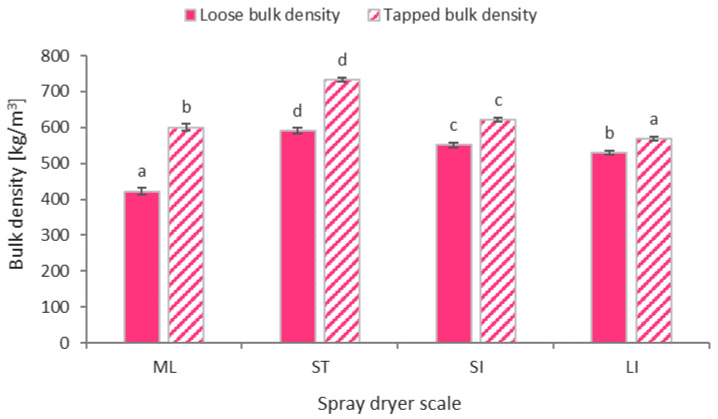
Loose and tapped bulk density of spray dried beetroot powder at different process scale (ML—mini-laboratory scale, ST—semi-technical scale, SI—small industrial scale, LI—large industrial scale; a, b, c, d—different letters show significant differences between spray dryer scale (*p* ≤ 0.05)).

The values of loose and tapped bulk densities are shown in Figure 4. Loose bulk density values were obtained in the range 422–591 kg/m^3^, and tapped bulk density values were obtained in the range 569–733 kg/m^3^. In the case of the centrifugal spray dryer series (ST, SI, LI), the use of a larger dryer resulted in lower bulk density values, which was confirmed by statistical analysis. In the case of the mini-laboratory-scale (ML) dryer, the values that were obtained show different characteristics, in particular, a much lower value of loose bulk density was recorded in relation to the other dryer sizes. This is due to the different liquid atomization system. The pneumatic two-fluid nozzle can aerate the dryer feed solution to some extent and can create powders with encapsulated air, which consequently leads to lower bulk densities. Other researchers have obtained loose bulk densities of spray-dried beetroot juice with maltodextrin as a carrier at the laboratory and semi-technical scales of 580 kg/m^3^ (Jedlińska et al. [25]), 410–615 kg/m^3^ (Janiszewska et al. [29]), 309–325 kg/m^3^ (Kapoor et al. [32]), and 616–698 kg/m^3^ (Gawałek et al. [33]) and a tapped bulk density at values of 730 kg/m^3^ (Jedlińska et al. [25]), 639–721 kg/m^3^ (Singh et al. [26]), 410–615 kg/m^3^ (Janiszewska et al. [29]), 352–389 kg/m^3^ (Kapoor et al. [32]), and 692–938 kg/m^3^ (Gawałek et al. [33]).

Comparing the differences between the loose and tapped bulk density values for each spray-drying scale, a decrease in these differences was observed with increasing dryer size, regardless of the liquid atomization system. This is directly reflected in the Hausner ratio (HR) values, which are measures of powder bulkiness. Table 2 shows the values of the HR and the angle of repose (AR), which is also a measure of powder flowability. Both of the powder flowability indices showed a statistically significant improvement in flowability when the spray dryer that was used increased in size. This dependence is related to the powder particle size (Table 1). Larger particle sizes improve the flowability of spray-dried beetroot powders. This is in line with Thomson’s [34] conclusion that a lower particle size generally provides lower flowability. In Table 2, each value of the flowability index (HR, AR) was also assigned a specific degree of flowability according to Carr’s classification [35]. The HR index showed a poorer flowability rating compared to the AR index. According to the HR values, beetroot powders with good or excellent flowability are only observed for industrial drying-scale-sized dryers (SI, LI), while according to the AR index, the already-tested semi-technical scale (ST) achieves good beetroot powder flowability. Similar differences in the powder flowability estimation using the loose and tapped bulk density method (HR) and the angle of repose method (AR) have been obtained by other authors: Kapoor et al. [32], Sarabandi et al. [36], and Dadi et al. [37] for spray-dried beetroot, apple, and moringa juices, respectively. Analyzing the obtained values of the flowability indices of spray-dried beetroot powders by other authors, Kapoor et al. [32] obtained beetroot powders with better flowability (HR = 1.25, AR = 33.83) at the laboratory scale with similar drying parameters. On a semi-technical scale, Gawałek et al. [33] obtained better powder flowability by comparing the Hausner coefficient (HR = 1.13) for the same drying conditions, while slightly worse flowability was obtained by using the angle of repose as a criterion (AR = 33°). However, a higher content of maltodextrin (70% d.m.) was used in this study. Jedlińska et al. [25] used a lower content of maltodextrin as a carrier (about 33% d.m.) in a semi-technical-scale experiment with dehumidified air and obtained a similar HR level (1.26) to the value found in the beetroot powder from the ST spray dryer.

### 2.6. Color

When producing colorants originating from plants, a very important aspect is to obtain a proper and reproducible color scale, i.e., to obtain a color with specific attributes: brightness, hue, and saturation. The CIE Lab system color scale of aqueous solutions containing manufactured beetroot powders was tested. The results are summarized in Table 3. The brightness (L*) of the beetroot powders produced in the centrifugal spray dryers (ST, SI, LI) showed no statistical differences (*p* ≤ 0.05), and only the beetroot powder from the ML dryer showed higher brightness. The larger size of the spray dryer affected the beetroot powder color changes in the red (increase in a* value) and blue (decrease in b* value) directions. The total color differences ΔE of the aqueous solutions of the beetroot powders with respect to the color of the solution prepared directly from the liquid juice concentrate showed a significant statistical decrease (*p* ≤ 0.05) when the size of the dryer increased. This implies that spray drying beetroot juice on a larger scale enables color changes that can occur during drying to be minimized. It should be noted that the ΔE values for all of the beetroot powders are not large and that the differences for both industrial scales (SI and LI) are practically imperceptible to the human eye.

The above correlations correspond to changes in other parameters, which confirm smaller thermal degradation impacts for larger dryers.

### 2.7. Violet Betalain Content

The content of betalain, i.e., violet and yellow pigments, specifically the percentage of their retention in the spray-drying process, is a very important performance parameter when considering beetroot powders. It directly affects their coloring power when these powders are used as natural food colorants. The spray-drying process, due to thermal processes that occur during them, degrades these pigments by several to tens of percent depending on the process conditions [23,25,27,29,31,33,38]. Degradation mechanisms such as isomerization, decarboxylation, or cleavage under heat and acidic environments can be different [26,39]. Therefore, in industrial practice, spray-drying process parameters are optimized for the retention (preservation) of betalain pigments. If beetroot juices are used in industrial production as natural colorants, they are standardized to the content of violet betalain pigments, which can be calculated in terms of betanin (BT).

**Figure 5:**
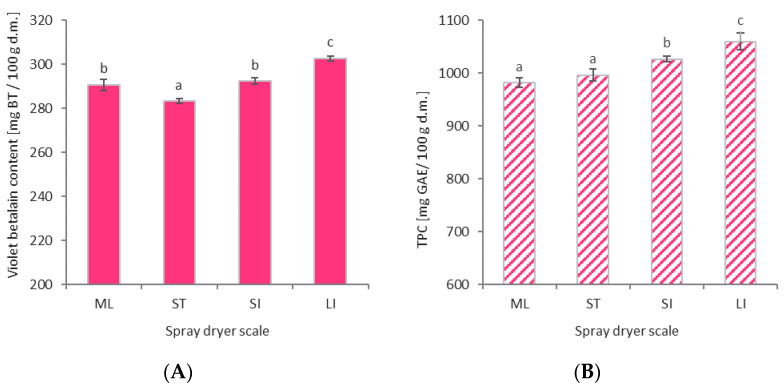
Violet betalain content (**A**) and total polyphenol content (TPC) (**B**) of spray-dried beetroot juice at different process scale (ML—mini-laboratory scale, ST—semi-technical scale, SI—small industrial scale, LI—large industrial scale); BT—betanin; GAE—gallic acid equivalent; d.m.—dry matter; a, b, c—different letters show significant differences between spray dryer scale (*p* ≤ 0.05).

In the present study, the content of violet betalain pigments in the obtained beetroot powders was obtained in the range of 283.3–302.7 mg/100 g, which represents a retention rate in the drying process of 91.4–97.6%, respectively. Other researchers who have considered the spray drying of beetroot juice have achieved betalain retention values of 61–70% (Bazaria et al. [23]), 91.6% (Do Carmo et al. [31]), and 34–88.5% (Ochoa et al. [38]) at the laboratory scale, while at the semi-technical scale, values of 26.7–29.3% (Janiszewska et al. [29]), 68–76% (Janiszewska [27]), and 85.5–95.5% (Gawałek et al. [33]) have been achieved. The size of the spray dryer that was used was found to be a factor causing statistically significant differences (*p* ≤ 0.05) in the content of violet betalain pigments (Figure 5A). For a range of centrifugal liquid spray dryers (ST, SI, LI), the use of a larger dryer resulted in higher levels of pigment retention. This dependence is related to the particle powder size, flowability, and powder yield. The lower stickiness of beetroot juice results in a higher yield of beetroot powder in the spray-drying process because larger particles with better flowability are formed. As a consequence, less beetroot powder remains in the drying system, and the thermal degradation of violet betalain pigments is reduced. From the above experimental results, it can be seen that by designing the drying process on a semi-technical scale and by increasing the scale, a significant reduction in the loss of violet betalain pigments can be expected. Increasing the scale from semi-technical (ST) to small industrial (SI), i.e., a sevenfold scale enlargement (7:1), resulted in a decrease in violet betalain pigments losses ranging from 9% to 6%. On the other hand, when the scale upsizing was much larger, i.e., fifty times (50:1), from semi-technical scale (ST) to industrial large scale (LI), the reduction in violet betalain pigment loss was even more significant. In this case, there was a decrease from 9% to 2%. For the laboratory dryer (ML), a higher value was achieved than would be expected due to the small scale; specifically, the expected values were similar to those achieved in the small industrial-scale (SI) dryer. This is due to the fact that the two-fluid nozzle atomization system results in milder drying thermal conditions than centrifugal atomizers despite using the same drying air inlet temperatures (T = 175 °C). The additional cool compressed air that transports and sprays the liquid raw material into the dryer chamber is responsible for this. In addition, the much higher ratio of the drying air to volume (Table 4) for the ML dryer results in the beetroot powder particles having a shorter residence time in the dryer.

### 2.8. Total Polyphenol Content (TPC)

Beetroot juice has bioactive properties in addition to coloring properties; hence, their retention (preservation) during spray drying was also investigated at different scales. Phenolic compounds such as phenolic acids and their derivatives (ferulic, vanillic, ellagic, caffeic, chlorogenic, p-coumaric, and sinapic acid) and flavonoids (quercetin, myricetin, kaempferol, rutin, vitexin, orientin, betagarin, betavulgarin, cochliophilin A, and dihydroisorhamnetin) can be found in beetroot juice [40,41,42,43]. Total polyphenol content (TPC) values were determined and ranged from 983–1058 mg GAE/100 g, representing retention rates in the range of 91.8–98.9%, respectively. In this case, the statistical significance of the differences between the different drying scales is much lower than that for the violet betalain pigment content, but a gentle increasing trend can be observed as the spray dryer scale increases (Figure 5B). The results obtained here may suggest that the polyphenols have a lower thermal sensitivity compared to the violet betalain pigments. TPC retention at a similar level (>90%) was also achieved during the spray drying of chokeberry juice [10,11] and bayberry juice [44]. In the case of non-optimal spray-drying conditions, lower TPC retention values can be obtained, similar to those for blueberry juice [45] and thyme extract [46]. There are also cases where the retention of TPC after the spray-drying process exceeds 100%. Such values were obtained by Zhang et al. [47] for cranberry juice (138–216%) and by Saikia et al. [48] for Khasi mandarin orange juice (417%). This effect is related to the possibility of changes in the chemical structure of phenolic compounds due to thermal treatment, which may improve the reactivity with the Folin–Ciocalteu reagent [49]. The intensity of these changes during spray drying depends on the phenolic profile of the dried material [47].

During the spray-drying of beetroot juice, betanin can be thermally decomposed into betalamic acid and cyclo-dopa-5-0-glycoside [50,51]. It is also possible to change the violet betalain pigments into yellow. Maillard reaction products may also be formed [52,53]. All of these compounds have antioxidative properties and can react with the Folin–Ciocalteu reagent to influence the TPC value. Therefore, during the spray-drying of beetroot juice, the temperature may cause a greater loss of violet pigments than the TPC values and antioxidative capacity can.

## 3. Materials and Methods

### 3.1. Materials

The research material was beetroot (*Beta vulgaris*) juice concentrate (SVZ International B.V., Breda, the Netherlands) with the following parameters: extract content 64.8 °Brix, violet betalain pigments content equal to 0.50%, density 1320 kg/m^3^ at 20 °C, pH 4.0, and 2.0 g/100 g citric acid. Potato maltodextrin with a dextrose equivalent of DE 11 (PEPEES S.A., Łomża, Poland) was used as a carrier. The same feed solution formulation was used for all of the drying processes: 34% solid content in water, 58.5% carrier content in dry mass. Assumptions were made to obtain the theoretical content of the violet betalain pigments at the level of 310 mg/kg at the moisture content of beetroot powder equal to 3%.

### 3.2. Spray Drying

Spray drying of the same solutions was carried out at different process scales: one laboratory-scale process, one semi-technical-scale process, and two industrial-scale processes (small and large). Table 4 summarizes the designations and data from the spray dryers that were used. A rotary atomizer system was used to spray the feed solution at the semi-technical scale (ST) and industrial scales (SI and LI). During rotary atomization, the feed is centrifugally accelerated to high velocity in the atomizer wheel before being discharged into the hot drying gas. At the laboratory scale (ML), however, atomization was performed using a two-fluid nozzle. In this system, atomization is achieved pneumatically by high-velocity compressed air making impact with the liquid feed.

In all of the drying processes, efforts were made to maintain comparable drying conditions/parameters. The principle that was adopted was to set a constant drying air inlet temperature (175 °C) and, by adjusting the flow rate of the feed solution (raw material), to maintain the outlet temperature in the range of 82–85 °C. In the case of centrifugal spray dryers (ST, SI, and LI), the same rotary atomizer speed was maintained at 15,000 rpm.

### 3.3. Powder Yield

Powder yield was calculated as the ratio of the dry matter content in the collected powder after each spray drying test process to the value of the dry matter content in the feed solution.

### 3.4. Microstructure of Particles

The beetroot powders were sputtered with gold and were examined for morphology using a scanning electron microscope SEM Zeiss Evo 40 (Carl Zeiss Microscopy Deutschland GmbH, Oberkochen, Germany; magnification: 500× and 1000×).

### 3.5. Particle Size Distribution

Particle size distribution was measured with a Mastersizer 2000 (Malvern Instruments Ltd., Malvern, UK) using laser diffraction. Isopropanol was used as a dispersant. Three percentiles (10th, 50th, and 90th), volume-weighted mean diameter D[4,3], and span (Equation (1)) of the volume distribution were determined.

(1) $$\mathrm{span}=\frac{D_{90}-D_{10}}{D_{50}}.$$

### 3.6. Moisture Content and Water Activity

The moisture content of the beetroot powder was analyzed using the oven method at 105 °C for 4 h. Water activity was measured in a Rotronic apparatus type Hygroscope DT (Rotronic AG, Bassersdorf, Switzerland) at 25 °C.

### 3.7. Bulk Density, Angle of Repose and Flowability

Loose bulk density (*ρ*_L_), tapped bulk density (*ρ*_T_), and the angle of repose (AR) were measured according to ASTM D6393 [54]. In addition to the natural angle of repose (AR), the Hausner ratio (HR) was used to evaluate the flowability of the beetroot powders. The Hausner coefficient was calculated as the ratio of tapped and loose bulk density: HR = *ρ*_L_/*ρ*_T_ [55].

### 3.8. Color

Using a Jasco V630 spectrophotometer (Japan), color parameters were determined using the CIE L*a*b* system for the 1% aqueous solutions of the resulting beetroot powders. The total color difference Δ*E* was also determined for all of the measurements (Equation (2)), where the solution prepared directly from the concentrate was considered as the standard.

(2) $$∆E=\sqrt{{\left(∆L\right)}^{2}+{\left(∆a\right)}^{2}+{\left(∆b\right)}^{2}}.$$

### 3.9. Violet Betalain Content

The content of the violet betalain pigments was determined using the method proposed by Nillson [56] with modifications, using a Jasco V630 spectrophotometer (JASCO International Co. Ltd., Tokyo, Japan). To 1 mL of test solution (1 g of beetroot powder + distilled water to a volume of 100 mL), 4 mL of pH 6.5 phosphate buffer was added. The absorbance was measured at 538 and 600 nm. The results were calculated in terms of betanin (BT) and were expressed as mg BT/100 g d.m. (dry matter) of beetroot powder. The retention of betalains in the spray-dried beetroot juice was calculated relative to the value measured in the feed solution before drying.

### 3.10. Total Polyphenol Content (TPC)

For the determination of the total polyphenol content (TPC), the Folin–Ciocalteu method, which has already been used in previous studies, was applied [11]. A sample (1 mL of solution: 0.5 g beetroot powder in 10 mL of 50% (*v*/*v*) methanol/water solution) was pipetted into a 100 mL volumetric flask and was diluted with distilled water. Determinations were performed using a Jasco V630 spectrophotometer (JASCO International Co. Ltd., Tokyo, Japan) by measuring absorbance at 765 nm. Results were expressed as mg gallic acid equivalent (GAE) per 100 g d.m. of beetroot powder. TPC retention in the dried beetroot juice was calculated relative to the value measured in the feed solution before drying.

### 3.11. Statistical Analysis

All of the determined physicochemical parameters were average values and were determined from measurements made after a min. of three repetitions. The statistical significance (or not) of the effect of the spray dryer-scale size on the physicochemical parameters of the beetroot powder and process efficiency was verified using one-way ANOVA analysis of variance with Tukey’s HSD test at a significance level of 0.05. Statgraphics 13.1 program was used.

## 4. Conclusions

Conducting spray drying experiments of fruit and vegetable juices at the experimental scale is necessary to determine the optimal drying conditions and to select the optimal carriers and solution formulations for industrial scale drying. Industrial process design for centrifugal spray dryers is definitely more efficient than using a semi-technical scale with the same spray system than using a laboratory-scale dryer with a two-fluid nozzle. The laboratory-scale dryer is more convenient for research, but many properties of the beetroot powder obtained on it do not correlate with the properties of beetroot powders obtained on the industrial scale. Among other things, the particle size distribution, microstructure, bulk density, and flowability of the powder are very different. This effect is caused not only by the difference in the feed solution spraying systems, but also by the large difference in the residence time of beetroot juice in the drying chamber. The larger scale of the spray-drying process at the same drying parameters makes it possible to obtain beetroot powders with a larger particle size, better flowability, color that is more shifted towards red and blue, and the higher retention of violet betalain pigments and polyphenols. The preservation of the colouring and bioactive properties in the spray-dried beetroot powder are related to the particle powder size, flowability, and powder yield. The lower stickiness of the beetroot juice results in a higher powder yield in the spray-drying process because larger particles with better flowability are formed. As a consequence, less beetroot powder remains in the drying system, and the thermal degradation of violet betalain pigments is reduced. During the spray-drying of beetroot juice, the temperature caused a greater loss of violet pigments than polyphenols. In both cases, retention above 90% was achieved at all spray-drying scales. As the size of the spray dryer increases, the efficiency of the process expressed in powder yield also increases. To obtain a powder yield >90% on the industrial scale, the process conditions should be selected so as to obtain a powder yield of a min. of 50% at a laboratory scale or 80% at a semi-technical scale.

## Figures and Tables

**Table 1:** Particle size distribution (mean ± standard deviation of five replications of D_10_, D_50_, D_90_, and D[4,3], span) of spray dried beetroot powders.

Spray Dryer Scale	D_10_ (μm)	D_50_ (μm)	D_90_ (μm)	D[4,3] (μm)	Span
ML	0.58 ± 0.02 ^a^	8.8 ± 0.4 ^a^	27.3 ± 0.7 ^a^	11.8 ± 0.4 ^a^	3.03 ± 0.05 ^c^
ST	0.91 ± 0.07 ^ab^	32.4 ± 1.1 ^b^	75.0 ± 1.9 ^b^	36.1 ± 1.3 ^b^	2.19 ± 0.02 ^b^
SI	1.10 ± 0.03 ^b^	37.6 ± 0.6 ^c^	75.4 ± 0.5 ^bc^	40.1 ± 0.3 ^bc^	1.98 ± 0.03 ^a^
LI	1.05 ± 0.19 ^b^	40.9 ± 1.3 ^d^	80.0 ± 1.5 ^c^	43.2 ± 1.7 ^c^	1.93 ± 0.03 ^a^

ML—mini-laboratory, ST—semi-technical, SI—small industrial, LI—large industrial, ^a^, ^b^, ^c^, ^d^ Different letters in the same column show significant differences (*p* ≤ 0.05).

**Table 2:** Characteristics of spray-dried beetroot powders flowability: mean ± standard deviation of three replications of Hausner ratio (HR) and angle of repose (AR).

Spray Dryer Scale	Hausner Ratio		Angle of Repose	
	(-)	Flowability *	(°)	Flowability *
ML	1.42 ± 0.01 ^d^	poor	42.2 ± 2.8 ^d^	passable
ST	1.24 ± 0.01 ^c^	fair	31.3 ± 1.9 ^c^	good
SI	1.13 ± 0.00 ^b^	good	22.6 ± 1.2 ^b^	excellent
LI	1.07 ± 0.00 ^a^	excellent	15.1 ± 0.9 ^a^	excellent

ML—mini-laboratory, ST—semi-technical, SI—small industrial, LI—large industrial, ^a^, ^b^, ^c^, ^d^ Different letters in the same column show significant differences (*p* ≤ 0.05), * Flowability of powders according to Carr’s [35] classification.

**Table 3:** Results for color parameters of 1% aqueous solutions of beetroot powders (mean ± standard deviation of 3 replications).

Spray Dryer Scale	L*	a*	b*	ΔE
ML	34.3 ± 0.3 ^a^	66.8 ± 0.4 ^a^	36.2 ± 0.3 ^d^	5.0 ± 0.3 ^d^
ST	35.6 ± 0.4 ^b^	67.9 ± 0.4 ^b^	35.8 ± 0.2 ^c^	3.6 ± 0.2 ^c^
SI	35.5 ± 0.3 ^b^	68.4 ± 0.2 ^b^	34.1 ± 0.2 ^b^	2.2 ± 0.1 ^b^
LI	36.4 ± 0.3 ^b^	69.7 ± 0.4 ^c^	33.4 ± 0.3 ^a^	0.6 ± 0.2 ^a^

ML—mini-laboratory, ST—semi-technical, SI—small industrial, LI—large industrial, ^a^, ^b^, ^c^, ^d^ Different letters in the same column show significant differences (*p* ≤ 0.05).

**Table 4:** Designations and data of spray dryers used.

Data/Parameters	Spray Dryers			
Designations	ML	ST	SI	LI
Scale	mini-laboratory spray dryer	semi-technical spray dryer	small industrial spray dryer	large industrial spray dryer
Drying chamber volume, m^3^	0.013	1.4	57	179
Water evaporation capacity, kg H_2_O/h	1	15	100	700
Liquid spray system	two-fluid nozzle	rotary atomizer	rotary atomizer	rotary atomizer
Spray nozzle/rotary disc diameter, mm	0.7	120	160	210
Batch size, kg	0.4	5	800	5000
Ratio of the drying air flow to the dryer volume, m^3^/m^3^·h	2692	335	87	81
Producer	Büchi Labortechnik AG, Flawil, Switzerland	Niro Atomizer, Søbork, Denmark	Combined—no name	Niro Atomizer, Søbork, Denmark
Type	B-290	FU 11 DA	-	C

## Data Availability

All data created and analyzed during the experiments were presented in this study.

## Sample Availability

Samples of the compounds are available from the author.
